# Managing Intracranial Pressure Crisis

**DOI:** 10.1007/s11910-024-01392-5

**Published:** 2024-12-19

**Authors:** Tanuwong Viarasilpa

**Affiliations:** Division of Critical Care, Department of Medicine, Siriraj Hospital, Mahidol University, 2 Wanglang Road, Bangkok, 10700 Thailand

**Keywords:** Intracranial pressure crisis, ICP, Intracranial hypertension, Management, Critical care, Neuromonitoring

## Abstract

**Purpose of Review:**

The objective of this review is to provide a comprehensive management protocol for the treatment of intracranial pressure (ICP) crises based on the latest evidence.

**Recent Findings:**

The review discusses updated information on various aspects of critical care management in patients experiencing ICP crises, including mechanical ventilation, fluid therapy, hemoglobin targets, and hypertonic saline infusion, the advantages of ICP monitoring, the critical ICP threshold, and bedside neuromonitoring.

**Summary:**

All aspects of critical care treatment, including hemodynamic and respiratory support and adjustment of ICP reduction therapy, may impact patient outcomes. ICP monitoring allows ICP values, trends, waveforms, and CPP calculation, which are helpful to guide patient care. Advanced neuromonitoring devices are available at the bedside to diagnose impaired intracranial compliance and intracranial hypertension, assess brain function, and optimize cerebral perfusion. Future research should focus on developing appropriate intervention protocols for both invasive and noninvasive neuromonitoring in managing ICP crisis patients.

## Introduction

Management of intracranial pressure (ICP) crisis varies between institutions depending on the availability of ICP monitoring [1–4]. The fourth edition of the Brain Trauma Foundation (BTF) Guidelines for the Management of Severe Traumatic Brain Injury (TBI) and The Seattle International Severe Traumatic Brain Injury Consensus Conference (SIBICC) management algorithm for patients with ICP monitors have been applied for the treatment of ICP crises in severe brain injuries, not limited to TBI [2–4]. However, not every patient with clinically suspected intracranial hypertension has immediate access to the ICP monitor, and many of these patients are managed without ICP monitors [1, 5, 6].

A large randomized controlled trial (RCT) reported no significant difference in the functional outcome between severe TBI patients managed with and without ICP monitoring. In that study, the Imaging and Clinical Examination (ICE) Protocol was used in the unmonitored group [7]. The ICE protocol has recently been revised to the Consensus-based management protocol (Consensus REVised ICE, CREVICE) for the treatment of severe TBI without ICP monitoring [8].

In recent years, there has been increased knowledge of management and neuromonitoring for acute brain injury patients. This review combines the SIBICC and CREVICE protocol and updates the latest information on critical care management and neuromonitoring in ICP crisis patients.

### Initial Management for ICP Crisis Patients

All patients with severe brain injury resulting in any form of cerebral edema, space-occupying lesions, or hydrocephalus are at risk for ICP crisis. These patients require immediate treatment of the specific cause of intracranial hypertension and frequent neurological examinations to evaluate the level of consciousness, pupillary size and reactivity, and new focal neurologic deficits. They should be initially managed according to the baseline measures (Tier zero) of the SIBICC recommendations in the intensive care unit (ICU; Table 1) [4, 9].

**Table 1:** Critical care management for patients with intracranial pressure crisis [4, 8–18]

Comatose brain injury patients with significant cerebral edema, space-occupying lesion, or hydrocephalus (GCS ≤ 8) are suspicious of intracranial hypertension	
**Baseline critical care measures (Tier zero)**	
• Admission to the intensive care unit • Treatment of the specific cause • Mechanical ventilation with lung-protective ventilation strategy by limited TV 6–8 ml/kg predicted BW with plateau pressure < 30 cmH_2_O, FiO_2_ ≤ 60% and appropriate PEEP to achieve SpO_2_ ≥ 94%, PaCO_2_ 35–38 mmHg, avoid hyperoxemia • Intravenous 0.9%NaCl to maintain euvolemia, and avoid fluid overload • Hemodynamic resuscitation to achieve mean arterial pressure ≥ 80 mmHg with adequate systemic organ perfusion, and avoid vasopressor complications • Head of the bed elevation to 30–45 degrees and head in the midline position • Fentanyl and propofol to target light sedation • Seizure prophylaxis for one week in patients with traumatic brain injury • Body temperature control < 37.5 °C • Blood glucose control to target 110–180 mg/dl • Hemoglobin target > 7 g/dl • Frequent neurological examinations to evaluate the level of consciousness, pupillary size and reactivity, and new focal neurologic deficits	
**Initiation of intracranial pressure lowering therapy** **(Tier one of the SIBICC and the CREVICE protocol)**	
• 20% mannitol 0.5–1 g/kg IV infusion over 5–15 min • 23.4%NaCl 30–60 ml or 3%NaCl 150 ml IV infusion over 10–30 min • Dexamethasone 10–20 mg IV bolus, followed by 4–24 mg/day for brain tumor or abscess • External ventricular drain placement for intraventricular hemorrhage or hydrocephalus • Surgical removal of the intracranial mass or hematoma or primary decompressive craniectomy when indicated	
**Clinical Neuroworsening**	
• Decrease in the motor GCS of ≥ 1 point • Decrease in pupillary reactivity • Pupillary asymmetry of > 2 mm • Bilateral pupillary dilatation • New focal motor deficit or abnormal posturing • Herniation syndrome or Cushing’s triad (hypertension, bradycardia, and slow respirations)	
**Adjustment of ICP-lowering treatment intensity without ICP monitoring**	**Adjustment of ICP-lowering treatment intensity with ICP monitoring**
Escalating the treatment intensity when • Develop clinical neuroworsening or • No improvement in clinical examination or brain imaging after initial treatment • Guided by noninvasive neuromonitoring	Escalating the treatment intensity when • Develop clinical neuroworsening • ICP ≥ 18–22 mmHg • ICP waveforms demonstrated reduced intracranial compliance • Guided by invasive and noninvasive neuromonitoring
**Tier 1 of the CREVICE protocol**	**Tier 1 of the SIBICC protocol**
• Scheduled infusion of hypertonic saline or 20% mannitol or both every 4 h	• Increase the dose and frequency of hypertonic saline or 20% mannitol or both to maintain ICP < 18–22 mmHg and normal ICP waveforms • Deep sedation • Maintain CPP target 60–70 mmHg
**Tier 2 of the CREVICE protocol**	**Tier 2 of the SIBICC protocol**
• Continuous infusion of 3% NaCl to increase serum sodium level • Hyperventilation to target PaCO_2_ of 30–35 mmHg • Deep sedation	• Hyperventilation to target PaCO_2_ of 32–35 mmHg • Mean arterial pressure challenge to assess static cerebral pressure autoregulation • Trial dose of neuromuscular blocking agents
**Tier 3 of the CREVICE and SIBICC protocol**	
• Barbiturate coma to target burst suppression on EEG • Mild hypothermia (35–36 °C) • Secondary decompressive craniectomy	

Abbreviations: GCS = Glasgow Coma Scale, TV = tidal volume, BW = body weight, FiO_2_ = fraction of inspiratory oxygen concentration, PEEP = positive end-expiratory pressure, SpO2 = oxygen saturation, PaCO2 = arterial partial pressure of carbon dioxide, SIBICC = The Seattle International Severe Traumatic Brain Injury Consensus Conference management algorithm for patients with ICP monitors, CREVICE = the Consensus-based management protocol for the treatment of severe TBI without ICP monitoring, NaCl = sodium chloride, IV = intravenous, ICP = intracranial pressure, EEG = electroencephalography

Systemic abnormalities resulting in secondary brain injury include shock/hypotension, oxygen and carbon dioxide abnormalities, hyponatremia, anemia, dysglycemia, sepsis, fever, agitation, and paroxysmal sympathetic hyperactivity [10, 19]. The occurrence of seizures and status epilepticus is an important intracranial cause of secondary brain injury other than elevated ICP.

### Respiratory Support

Patients who are unable to maintain airway patency or have Glasgow Coma Scale (GCS) ≤ 8, or those with respiratory failure should be intubated and mechanically ventilated to achieve adequate oxygen saturation (SpO_2_ ≥ 94%) by avoiding hyperoxemia and maintain arterial partial pressure of carbon dioxide (PaCO_2_) at 35–38 mmHg to prevent cerebral vasoconstriction or vasodilatation [4, 11, 12].

Although the high-quality evidence for optimal mechanical ventilation strategies in patients with ICP elevation is limited, prior studies suggested that lung-protective ventilation strategies with a low tidal volume of 6–8 ml/kg predicted body weight and positive end-expiratory pressure of 5–8 cmH_2_O may be safely employed in acute brain injury patients [12–15].

### Hemodynamic Stabilization

Intravenous isotonic crystalloids should be given to maintain euvolemia and ensure adequate systemic and cerebral perfusions unless there is evidence of hypervolemia; 0.9% sodium chloride is a preferred crystalloid [16]. Critically ill TBI patients with TBI receiving balanced crystalloids had a higher 90-day mortality and were less likely to be discharged home compared with those receiving 0.9% sodium chloride solutions in subgroup analyses of two RCTs [17, 18].

The initial blood pressure target for severe TBI patients varies between guidelines. The BTF guidelines recommend targeting systolic blood pressure ≥ 100 mmHg for patients aged 50–69 years or ≥ 110 mmHg for patients aged < 50 or > 70 years, and CPP of 60–70 mmHg corresponding to the SIBICC recommendation [2, 4], while the CREVICE protocol suggests an initial mean arterial pressure (MAP) target of ≥ 90 mmHg for patients without ICP monitoring [8]. The blood pressure target for acute brain injury other than TBI refers to standard guidelines for individual diseases [20–22]. The initial MAP target of > 80 mmHg is recommended in the NeuroVanguard approach for taking care of brain-injured patients [10].

Data on the patient’s mean arterial blood pressure (MAP), the amount of fluid accumulation, urine output, blood lactate, central venous oxygen saturation, capillary refill time, level of consciousness, and cardiac output and neuromonitoring, when available, should be integrated to determine resuscitation endpoint and guide fluid therapy [16, 23, 24].

### Supportive Measures to Prevent ICP Elevation

Other supportive measures to prevent ICP elevation include elevating the head of the bed to 30–45 degrees and keeping the head in the midline position to enhance cerebral venous return, pain and agitation control with fentanyl and propofol titrating to target light sedation assessed by the Richmond Agitation-Sedation Scale (RASS) score of 0 to −2 [25–27], early seizure prophylaxis for one week in TBI patients with phenytoin or levetiracetam, and fever control by targeting body temperature of < 37.5 °C [4].

### Other Critical Care Measures

Blood glucose should be controlled within ranges of 110–180 mg/dl since tight glycemic control (blood glucose < 110 mg/dl) increases the risk of cerebral metabolic crisis [19, 28]. Hemoglobin (Hb) level should be initially targeted at > 7 g/dl since there is no difference in the unfavorable outcomes between TBI patients receiving liberal (transfusion when Hb ≤ 10 g/dl) or restrictive (transfusion when Hb ≤ 7 g/dl) red blood cell transfusion strategy in a recent RCT (68.4% vs 73.5%; relative risk [RR], 0.93; 95%CI, 0.83–1.04) [29].

### Initiation of ICP-Lowering Therapy

The primary goal for the management of intracranial hypertension includes preventing brain herniation and cerebral ischemia. Patients with a decreased level of consciousness who have a cisternal compression, midline shift, or unevacuated mass lesion on brain imaging, or herniation syndromes are suspicions of intracranial hypertension; the ICP-lowering therapies should be initiated (Tier one of the SIBICC recommendation and the CREVICE protocol), and early neurosurgery consultation is recommended [4, 8, 30, 31].

The selection between hypertonic saline and mannitol depends on the patient's condition and center preference. A recent cohort study evaluating TBI patients receiving mannitol or hypertonic saline treatment in the ICU (CENTER-TBI) found no differences in the ICU mortality (odds ratio [OR], 1.0; 95%CI, 0.4–2.2) and Extended Glasgow Outcome Scale (GOSE) at 6 months (OR, 0.9; 95%CI, 0.5–1.6) between the two groups [32].

Mannitol is an osmotic diuretic that may cause hypotension from intravascular volume depletion, hypokalemia, and metabolic alkalosis. Other adverse effects of mannitol include hyperglycemia as it is a sugar alcohol and acute kidney injury in patients receiving mannitol treatment > 200 g/day [33, 34]. The recommended initial dose of 20% mannitol for emergency treatment of ICP crisis is 0.5–1 g/kg intravenous infusion over 5–15 min.

Hypertonic saline results in intravascular volume expansion and increased serum osmolarity; thus, it may cause cardiogenic pulmonary edema. Other adverse effects of hypertonic saline include hypernatremia, hyperchloremic metabolic acidosis, and osmotic demyelination syndrome. The given amount of hypertonic saline depends on the sodium concentration in the solution: 150 ml of 3%NaCl, 100 ml of 5%NaCl, 75 ml of 7.5%NaCl, or 30–60 ml of 23.4%NaCl can be administered intravenously over 10–30 min. Hypertonic solutions with a concentration of > 7.5% should be given through a central venous catheter [3, 9, 33, 35]. Dose adjustment for mannitol or hypertonic saline depends on the patient's symptoms, brain imaging, and ICP and neuromonitoring data.

Corticosteroids can be considered as an adjunctive treatment in patients with perilesional edema from brain tumors or abscesses [3, 36, 37]. Although there is limited data on the dosage and type of corticosteroids in this condition, dexamethasone is the most commonly used with a suggested dose of 10–20 mg bolus intravenously, followed by 4–24 mg/day, divided into 2–4 times daily [3, 36].

External ventricular drain (EVD) should be placed in patients with intraventricular hemorrhage or hydrocephalus [22, 35]. Surgical removal of the intracranial mass or hematoma or surgical decompression should be performed when indicated [20, 21, 38–41].

### Clinical Neuroworsening

Clinical neuroworsening is defined as a decrease in the motor GCS of ≥ 1 point, a new decrease in pupillary reactivity, pupillary asymmetry of > 2 mm, bilateral pupillary dilatation, a new focal motor deficit or abnormal posturing, a herniation syndrome, or Cushing’s triad [3, 4, 8].

Patients with clinical neuroworsening should be re-evaluated for both extracranial and intracranial etiologies by reassessing physiological parameters, including ICP, MAP, cerebral perfusion pressure (CPP), SpO_2_, end-tidal CO2, and body temperature, and performing a blood test for serum glucose, complete blood count, blood chemistry, arterial blood gas, septic work up and emergency computed tomography (CT) of the brain [4, 8].

### Adjustment of Intracranial Pressure-Lowering Therapies without ICP Monitoring

When no ICP monitor is available, either in resource-limit settings or while awaiting ICP insertion, escalating ICP-lowering treatments should be considered when patients develop new clinical neuroworsening or have no improvement in clinical examination or brain imaging after initial treatment. The recommended time for the follow-up brain imaging is 24 and 48 h after the onset of the brain injury and whenever the patients have clinical neuroworsening [8].

The CREVICE protocol recommends a three-tier stepwise approach for ICP treatment (Table 1), starting with the scheduled infusion of hypertonic saline or mannitol every 4 h [8]. Hyperosmolar therapy with hypertonic saline and mannitol can be administered in combination in severe cases. A serum sodium of ≥ 155 mEq/L, serum osmolarity of ≥ 320 mEq/L, or osmolar gap of ≥ 20 mEq/L are sometimes used as a limit for hypertonic saline and mannitol administration, although it is clear that additional dosing can continue to lower ICP even after these thresholds have been exceeded (tier one).

For patients with ongoing clinical neuroworsening, continuous infusion of 3% NaCl to increase serum sodium level, hyperventilation to target PaCO_2_ of 30–35 mmHg, or increasing dose of sedation can be considered (tier two). Although continuous hypertonic saline infusion did not improve functional outcome and mortality at 6 months in patients with moderate to severe TBI in a randomized controlled trial, only 35% of the study population had documented intracranial hypertension, and 72% had severe TBI (GCS ≤ 8) [42]. Treatment with continuous hypertonic saline infusion was associated with reduced risk of mortality, intracranial hypertension, and unfavorable outcomes in patients with acute brain injury in prior cohort and meta-analysis studies [43, 44].

In refractory cases, secondary decompressive craniectomy, barbiturate coma with high-dose thiopental, or pentobarbital titrating to target burst-suppression on continuous electroencephalography monitoring, or mild hypothermia with a core temperature of 35–36 °C can be used (tier three) [3, 8, 45, 46].

A prior cohort study reported that severe TBI patients without ICP monitoring who were treated with the CREVICE protocol had better functional outcomes compared with those managed without standard protocol [47]. However, the CREVICE protocol is expected to be a treatment guideline for the management of ICP crises in resource-limit settings; it is not intended to replace management with ICP monitoring because the changes in the patient's clinical examination or brain imaging may be more delayed than ICP changes, leading to an inaccurate adjustment of the ICP-lower therapy, and no CPP calculation allows [8]. In centers where ICP monitoring is available, the treatment for ICP crisis should be guided by the ICP data.

### Adjustment of Intracranial Pressure-Lowering Therapies guided by ICP Monitoring

An excellent review of the ICP physiology and monitoring technique has recently been published [31]. Although the benefits of ICP monitoring on patient outcomes have not been clearly demonstrated, there are important advantages of ICP monitoring [5, 7, 48, 49]. The ICP values and trends, ICP waveforms, and CPP calculation are crucial information to guide patient management. The ICP-lowering therapies can be provided promptly when the ICP values are above the treatment threshold or when the P2 (tidal wave) peak amplitude of the ICP waveforms is equal to or higher than P1 (percussion wave), indicating reduced intracranial compliance; this may allow faster escalation of the treatment intensity, which has been shown to be associated with reduced mortality in acute brain injury patients [1, 3, 9, 50].

Another advantage of ICP monitoring is that dynamic cerebral autoregulation monitoring can be performed to individualize CPP targets when the ICP and MAP are continuously recorded, resulting in better optimization of brain perfusion [51, 52].

In general, ICP monitoring is indicated in patients who have severely decreased levels of consciousness (GCS ≤ 8), and brain imaging revealed brain herniation or pathology that may cause increased ICP, including hydrocephalus, SAH, ICH, cerebral edema, or significant space-occupying lesion from brain tumor or abscess [9, 53]. For TBI patients, ICP is recommended in those with GCS ≤ 8 and abnormal brain imaging, including intracranial hematoma, cerebral contusion, brain swelling or herniation, or basal cistern compression. The ICP monitoring should be considered in severe TBI patients (GCS ≤ 8) with normal brain CT who have at least two of the following criteria: age > 40 years, unilateral or bilateral motor posturing (GCS motor score of 2 or 3), and systolic blood pressure of < 90 mmHg since the ICP elevation may occur in these patients [2].

### Critical Threshold for The Intracranial Pressure

The critical ICP threshold is controversial. The ICP target of < 20 mmHg was recommended in the previous BTF guidelines [7]. The fourth edition of BTF guidelines and SIBICC recommendations target an ICP of < 22 mmHg based on one observational study designed to identify the ICP threshold for discriminating between the patients who survived or died and who had favorable or unfavorable outcomes [2, 4, 54]. However, a recent observational study reported an ICP threshold of ≥ 18 mmHg for both survival and favorable outcomes in TBI patients receiving ICP monitoring [55]. In a survey among neurocritical care professionals, only 28% of the participants followed the ICP threshold of ≥ 22 mmHg for adjusting the intensity of the ICP-lowering therapy, whereas 62% of the participants used a more flexible ICP threshold based on additional data from ICP waveforms and other neuromonitoring when the ICP value ranges from 18–22 mmHg [1].

### The SIBICC Algorithms for the Management Of Patients with ICP Monitors

When an ICP monitor is placed, the stepwise algorithm recommended by the SIBICC can be followed (Table 1). In tier one management, the dose and frequency of the hyperosmolar treatment with mannitol or hypertonic saline are adjusted to maintain the ICP values below the critical threshold and normal ICP waveforms. The depth of sedation can be increased until a RASS score of −3 to −5 (unarousable) is reached. CPP should be maintained within 60–70 mmHg [4].

In tier two management, hyperventilation to target PaCO_2_ of 32–35 mmHg can be used. A MAP Challenge can be performed in this tier to assess the status of static pressure autoregulation (sPAR) by observing the changes in ICP after increasing MAP and CPP. When sPAR status is intact, an increase in MAP and CPP by 10 mmHg for 20 min with fluid bolus and norepinephrine infusion will decrease ICP due to cerebral vasoconstriction and reduced cerebral blood volume. In this condition, if there is no other systemic complication of hemodynamic augmentation, the elevated MAP and CPP should be maintained at the level that ICP is lower than the critical threshold. However, when the sPAR status is disrupted, an increase in MAP and CPP during the MAP Challenge will increase cerebral blood flow and cause further ICP elevation; in this situation, the vasopressor infusion should be stopped to allow the MAP to return to baseline. Other ICP-lowering therapies, including mannitol, hypertonic saline, sedation, and EVD drainage, should not be adjusted during the MAP Challenge procedure for accurate interpretation. A trial dose of neuromuscular blocking agents may be considered [4].

The tier three management in the SIBICC algorithm includes surgical decompressive craniectomy, barbiturate coma, and mild hypothermia, similar to the CREVICE protocol. The dose of thiopental or pentobarbital will be increased until the ICP is lower than the critical threshold or burst suppression occurs on EEG monitoring [4].

### Bedside Neuromonitoring-Guided Intracranial Hypertension Treatment

Many invasive and noninvasive neuromonitoring modalities are available at the bedside (Table 2). The commonly used neuromonitoring techniques include noninvasive ICP waveform analysis, transcranial Doppler ultrasonography (TCD), optic nerve sheath diameter (ONSD), quantitative pupillometry, cerebral autoregulation monitoring, brain tissue oxygen tension (PbtO2) and cerebral microdialysis [10, 51, 52, 56].

**Table 2:** Bedside neuromonitoring [56–71]

Neuromonitoring devices	Monitoring objectives and threshold values
Noninvasive ICP waveform analysis	**Objective** • Assess intracranial compliance **Threshold value** • P2:P1 ≥ 1 or TTP ≥ 0.2 s indicates impaired intracranial compliance
Transcranial Doppler ultrasonography (TCD)	**Objective** • Predict and exclude intracranial hypertension **Threshold value** • TCD-estimated ICP < 20.5 mmHg can exclude intracranial hypertension
Optic nerve sheath diameter (ONSD)	**Objective** • Predict intracranial hypertension **Threshold value** • ONSD ≥ 5.3 mm had a good correlation with invasive ICP > 20 mmHg
Quantitative pupillometry	**Objective** • Assess the impact of ICP on brain function by the Neurological Pupil index **Threshold value** • NPi < 3 indicates decreased pupillary reactivity • Good correlation between NPi and ICP changes
Cerebral autoregulation monitoring	**Objective** • Individualize cerebral perfusion pressure target based on pressure reactivity index **Threshold value** • Zero or negative PRx value indicates an intact autoregulation status • Positive PRx value indicates cerebral autoregulation impairment
Brain tissue oxygen tension (PbtO_2_)	**Objective** • Monitor the balance between cerebral oxygen delivery and oxygen consumption **Threshold value** • PbtO_2_ < 20 mmHg indicates brain tissue hypoxia
Cerebral microdialysis	**Objective** Monitor cerebral metabolism • Cerebral ischemia • Mitochondrial dysfunction • Cerebral hypoglycemia **Threshold value** • Cerebral lactate > 4 mmol/L, pyruvate < 70 mmol/L, and LPR > 25 indicates cerebral ischemia • Cerebral lactate > 4 mmol/L, pyruvate > 70 mmol/L, and LPR > 25 indicates mitochondrial dysfunction • cerebral glucose < 0.8 mmol/L indicates cerebral hypoglycemia

Abbreviations: ICP = intracranial pressure, P2:P1 = the ratio of P2 (tidal wave) to P1 (percussion wave) amplitude of the ICP waveforms, TTP = time from the beginning to the peak amplitude of the ICP waveforms, TCD = transcranial Doppler ultrasonography, ONSD = optic nerve sheath diameter, NPi = Neurological Pupil index, PRx = pressure reactivity index, PbtO_2_ = brain tissue oxygen tension, LPR = lactate-to-pyruvate ratio

### Noninvasive ICP Waveform Analysis

Noninvasive ICP waveform (ICPW) analysis is a promising technique that uses a tension gauge sensor to measure the nanometer displacement of skull deformation resulting from changes in ICP [57, 72]. The sensor is attached to a headband that is placed around the patient’s head, with a sensor positioned at 3 cm above the anterior third of the orbitomeatal line [56]. It measures the amplitude of P2 and P1 of the ICPW and calculates the P2:P1 ratio and time from the beginning to the peak amplitude of the ICPW (time-to-peak, TTP). In normal circumstances, P2 amplitude is lower than P1; thus, the P2:P1 ratio is < 1. When intracranial compliance is impaired, the P2 amplitude increases higher than the P1, resulting in the P2:P1 ratio of ≥ 1, and TTP is longer than 0.2 s [56, 57]. A prior study reported a strong agreement for the P2:P1 ratio (κ agreement of 88%) and moderate agreement for the TTP value (κ agreement of 71%) between invasive and noninvasive ICPW analysis methods. The P2:P1 ratio can predict intracranial hypertension (area under the curve [AUC], 0.79; 95%CI, 0.72–0.93) better than the TTP value (AUC, 0.69; 95%CI, 0.60–0.74) [57].

Since no neurosurgical procedure is required, and multiple patients can reuse the sensor, this technique may be helpful in situations when invasive ICP monitoring is not accessible and can be used as a screening tool to select patients for ICP monitor placement [56].

### Transcranial Doppler Ultrasonography (TCD)

Cerebral blood flow (CBF) velocity detected by TCD, together with the patient's mean arterial blood pressure, can be used to estimate CPP and ICP noninvasively with a proposed mathematical formula [58, 73]. The most commonly used technique is applying a 2 MHz ultrasound probe over a temporal window of the scalp to measure the middle cerebral artery (MCA) blood flow [58].

A recent multicenter study demonstrated that the TCD-derived ICP (ICP_TCD_) threshold of ≥ 20.5 mmHg can predict the ICP of > 22 mmHg measured by intraventricular or intraparenchymal ICP devices (ICP_Invasive_) with a sensitivity of 70% (95%CI, 41–93%), specificity of 72% (95%CI, 52–94%), positive predictive value of 23% (95%CI, 16–50%), negative predictive value of 96% (95%CI, 93–99%), and accuracy of 72% (95%CI, 55–90%). With a high negative predictive value, intracranial hypertension (ICP_Invasive_ > 22 mmHg) can be ruled out when the ICP_TCD_ is < 20.5 mmHg [59].

### Optic Nerve Sheath Diameter (ONSD)

Since the optic nerve is part of the central nervous system extending into the orbit and is surrounded by the cerebrospinal fluid (CSF), the elevated ICP is transmitted to the CSF space around the optic nerve, resulting in increased ONSD [60]. The ONSD can be measured at 3 mm behind the retina using a 7.5 MHz linear ultrasound probe [61]. A recent study found that ONSD ≥ 5.3 mm had a good correlation with intracranial hypertension (ICP_Invasive_ > 20 mmHg), with an area under the curve (AUC) of 0.78 (95%CI, 0.68–0.88) [61].

### Quantitative Pupillometry

Quantitative pupillometry precisely measures pupillary size and reactivity using an infrared camera with calibrated light stimulation of fixed intensity and duration. It has an algorithm to calculate the Neurological Pupil Index (NPi) to quantify pupillary reactivity from the measured pupillary variables. The NPi should be assessed from both eyes; the NPi value ranges from 0 to 5: NPi of 0 indicates nonreactive pupils, NPi < 3 indicates decreased pupillary reactivity, and NPi 3–5 indicates normal pupillary reactivity [62, 63].

The changes in the NPi may reflect the impact of ICP on brain function since there is a good correlation between NPi and ICP changes. The NPi decreases during ICP elevation and increases after ICP reduction by hyperosmolar treatment [64]. These findings suggest that quantitative pupillometry may be a valuable tool for monitoring brain function in ICP crisis patients.

### Cerebral Autoregulation Monitoring

Cerebral autoregulation is the capability of cerebral vessels to alter cerebrovascular resistance in response to CPP change to maintain constant CBF [65]. The pressure reactivity index (PRx) is the commonly used autoregulation index calculated from the moving correlation coefficient between the changes in the ICP and MAP over a 5-min window. Zero or negative PRx value indicates an intact autoregulation status, whereas a positive PRx value indicates cerebral autoregulation impairment [51, 66]. Previous studies reported that a PRx threshold for impaired autoregulation ranging from + 0.05 to + 0.35 was associated with unfavorable outcomes and mortality in TBI patients; this threshold can be applied to determine the optimal ranges of CPP (CPP_opt_) between the lower limit (LLA) and upper limit of cerebral autoregulation (ULA) where the PRx can be maintained below the critical threshold [67].

In a feasibility study on the cerebral autoregulation-guided optimal CPP target in severe TBI patients, the CPP_opt_ was available for 77%, and the CPP could be controlled within the optimal range for 47% of the monitored time, which was higher than the feasibility criteria [74]. A phase III clinical study is required to evaluate its impact on clinical outcomes.

### Brain tissue Oxygen Tension (PbtO_2_)

Since brain tissue hypoxia commonly occurs despite no ICP elevation due to an imbalance between cerebral oxygen delivery and oxygen consumption, PbtO2-guided treatment has been proposed in attempting to improve patient outcomes [68, 75, 76]. The PbtO_2_ monitoring technique involves placing a small catheter through the skull into the brain parenchyma to measure PbtO_2_ from 15 mm^3^ of the brain volume around the catheter tip. The PbtO_2_ cut-off value for brain tissue hypoxia is < 20 mmHg [9, 69]. Cerebral oxygen delivery can be improved by optimizing intravascular volume, cardiac output, and CPP to increase CBF, adjusting mechanical ventilation to increase arterial oxygen saturation and maintain normal PaCO_2_, and providing red blood cell transfusion to increase Hb level. Conditions that might increase cerebral oxygen consumption, including seizures, status epilepticus, fever, and agitation, should be identified and treated [9, 68, 77].

A phase III RCT study involving 318 severe TBI patients with ICP monitoring reported no significant difference in the 6-month functional outcomes assessed by GOSE between patients treated with and without PbtO_2_ monitoring [78]. Unfavorable outcomes occurred in 52% of patients receiving both ICP and PbtO_2_ monitoring and 51% of those with ICP monitoring only (OR, 1.0; 95%CI, 0.6–1.7; P = 0.95) [78]. The limitation of this trial is that the study protocol used an increased fraction of inspired oxygen as an initial strategy to increase PbtO_2_, which is less effective in improving cerebral oxygen delivery than CBF optimization [79, 80]. Two larger multicenter RCTs designed to evaluate the efficacy of PbtO2-guided protocol in severe TBI patients are still ongoing (BOOST-3, NCT03754114; and BONANZA-GT, ACTRN12619001328167) [79, 81].

### Cerebral Microdialysis

Cerebral microdialysis requires a probe consisting of a dual-lumen channel and a semipermeable membrane inserted into the brain parenchyma to monitor cerebral lactate, pyruvate, glucose, and glutamate in the brain interstitium [70]. Cerebral microdialysis can be used with the PbtO_2_ to understand abnormal cerebral metabolism. Elevated cerebral lactate > 4 mmol/L with reduced cerebral pyruvate < 70 mmol/L, high lactate-to-pyruvate ratio (LPR) > 25, and PbtO_2_ < 20 mmHg indicates brain ischemia. In contrast, an increase in LPR and cerebral lactate with high cerebral pyruvate and normal PbtO_2_ suggests mitochondrial dysfunction. Cerebral glucose measured by microdialysis can be used to guide insulin therapy to prevent cerebral hypoglycemia (cerebral glucose < 0.8 mmol/L) [28, 71].

This monitoring technique is not widely used due to limited data on the appropriate treatment protocol guided by the biochemical results and the impact on patient outcomes in a clinical study [70, 82].

### Clinical Application on Patient Management (Fig. 1)

In ideal circumstances, ICP crisis patients should be managed under ICP-guided therapy with a flexible ICP threshold determined by the ICP waveforms and trend values. PbtO_2_ can be employed to assist cerebral oxygen delivery optimization. Cerebral autoregulation monitoring should be considered to personalize the optimal CPP target. Quantitative pupillometry can be used to assess brain function in comatose patients.

**Fig. 1:**
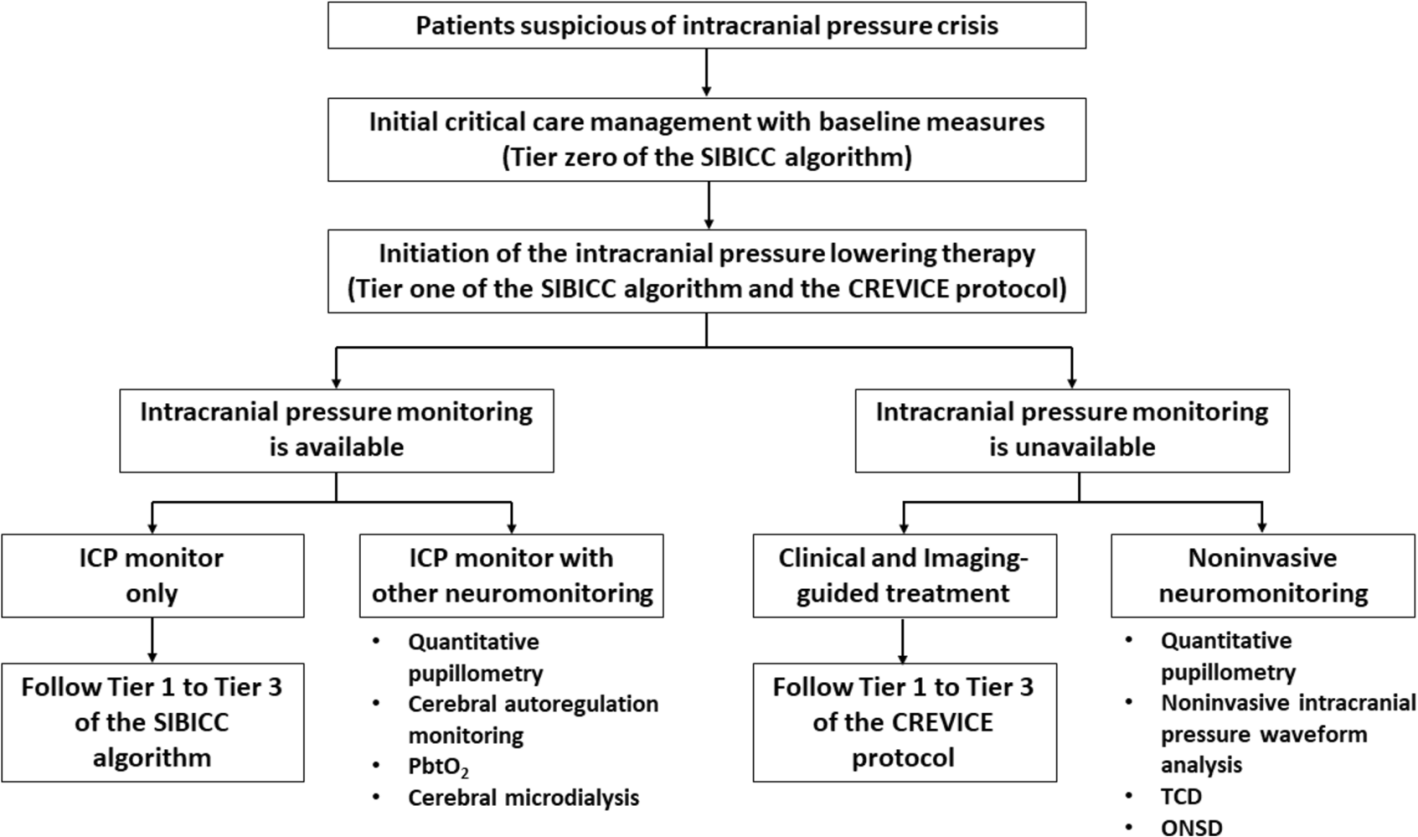
Framework for management of intracranial pressure crisis. The SIBICC algorithm = The Seattle International Severe Traumatic Brain Injury Consensus Conference management algorithm for patients with ICP monitors. The CREVICE protocol = the Consensus-based management protocol for the treatment of severe TBI without ICP monitoring. PbtO_2_ = brain tissue oxygen tension monitoring. TCD = Transcranial Doppler ultrasonography. ONSD = Optic nerve sheath diameter

In resource-limit settings, invasive neuromonitoring, including ICP and PbtO_2_ monitoring, is not feasible. Noninvasive monitoring techniques are beneficial. ONSD ultrasonography can be used to detect intracranial hypertension. TCD may be considered for ICP and CPP estimation. A noninvasive ICPW analysis device can be applied to recognize reduced intracranial compliance and guide the escalation of ICP-lowering therapy. Sequential NPi monitoring will be helpful in evaluating treatment response and detecting clinical neuroworsening in comatose patients.

## Conclusions

All aspects of critical care management, including appropriate hemodynamic, respiratory, and neurological support, are essential to achieve the best possible patient outcomes. The ICP reduction therapy should be initiated in patients with suspected intracranial hypertension regardless of the presence of ICP monitoring. The advantage of ICP monitoring is highlighted; the ICP values, trends, and waveforms should be interpreted together to adjust the treatment intensity. Various neuromonitoring devices are helpful in detecting impaired intracranial compliance and elevated ICP, assessing brain function, and optimizing cerebral perfusion.

## Source of Funding and Support

None.

## Data Availability

No datasets were generated or analysed during the current study.
